# A Hybrid Spiral Microfluidic Platform Coupled with Surface Acoustic Waves for Circulating Tumor Cell Sorting and Separation: A Numerical Study

**DOI:** 10.3390/bios12030171

**Published:** 2022-03-11

**Authors:** Rana Altay, Murat Kaya Yapici, Ali Koşar

**Affiliations:** 1 Faculty of Engineering and Natural Sciences, Sabanci University, Istanbul 34956, Turkey; raltay@sabanciuniv.edu (R.A.); mkyapici@sabanciuniv.edu (M.K.Y.); 2 Center of Excellence for Functional Surfaces and Interfaces for Nano-Diagnostics, Faculty of Engineering and Natural Sciences, Sabanci University, Istanbul 34956, Turkey

**Keywords:** cell separation, cell sorting, circulating tumor cells (CTCs), inertial microfluidics, surface acoustic wave (SAW), hybrid separation

## Abstract

The separation of circulating tumor cells (CTCs) from blood samples is crucial for the early diagnosis of cancer. During recent years, hybrid microfluidics platforms, consisting of both passive and active components, have been an emerging means for the label-free enrichment of circulating tumor cells due to their advantages such as multi-target cell processing with high efficiency and high sensitivity. In this study, spiral microchannels with different dimensions were coupled with surface acoustic waves (SAWs). Numerical simulations were conducted at different Reynolds numbers to analyze the performance of hybrid devices in the sorting and separation of CTCs from red blood cells (RBCs) and white blood cells (WBCs). Overall, in the first stage, the two-loop spiral microchannel structure allowed for the utilization of inertial forces for passive separation. In the second stage, SAWs were introduced to the device. Thus, five nodal pressure lines corresponding to the lateral position of the five outlets were generated. According to their physical properties, the cells were trapped and lined up on the corresponding nodal lines. The results showed that three different cell types (CTCs, RBCs, and WBCs) were successfully focused and collected from the different outlets of the microchannels by implementing the proposed multi-stage hybrid system.

## 1. Introduction

Cancer is one of the most pervasive and fatal diseases for humanity. Yet, microfluidic platforms can provide early cancer diagnosis, which is vital in the treatment of patients. According to the American Cancer Society, around 1.8 million new cancer cases were diagnosed in the United States of America in 2020, while the number of deaths was reported to be 606,520 [1]. The World Health Organization (WHO) reported that the number of deaths related to cancer can be reduced by up to 30% by diagnosing cancer before the metastasis stage [2].

Cancer involves an abnormal change of the cell’s shape and function due to defects in the genetic material or DNA, which differentiates these cells from healthy ones [3,4]. These modified cells experience cell proliferation, multiply rapidly in an uncontrolled manner, gather in groups, and form tumors. Cancer metastasis starts when tumor cells called circulating tumor cells (CTCs) spread through the body via the lymph and blood systems and invade other tissues that they colonize, forming secondary tumors [5]. It has been reported that metastasis is the main reason for at least two-thirds of the deaths of patients with cancer [6]. However, the isolation of CTCs from a blood sample enables further understanding of a patient’s health, since these cells carry different genomic and phenotypic properties with respect to primary tumor cells [7]. This has been the main motivation for many valuable studies in the literature conducted during the last decade. Microfluidics has been exploited in this regard for cancer diagnosis and treatment [8,9,10,11,12,13,14].

Microfluidic devices provide many advantages such as the consumption of a low amount of samples and reagents, which makes these devices preferable from the perspectives of cost, efficiency, rapidity of the analysis, and flexibility for their integration to lab-on-a-chip or organ-on-a-chip platforms. Since the number of CTCs (1–10/mL) in adult human blood with metastatic disease is very low compared to the number of white blood cells (5–10 × 10^6^/mL) and red blood cells (5–9 × 10^9^/mL) [15], the integration of these devices in cancer research mainly depends on the enrichment and separation of CTCs from a blood sample. For this purpose, several studies have been conducted involving the analysis of different particle sizes and density, which have helped develop methods for the isolation of CTCs from human blood samples [2,16,17,18,19,20].

Based on the need for an external source for operation, both label-free particle and cell separation studies have been mainly categorized into active and passive approaches. Active cell separation approaches utilize several external forces such as magnetic [21,22], electric [23,24], acoustic [25,26], and optical [27,28] forces to identify cells according to their response to applied fields or their physical characteristics such as size and density [4,14]. Passive approaches depend on the physical properties of the cells, which can be manipulated by inertial forces [29], filtration [30], deterministic lateral displacement [31], or adhesion [32]. The operation does not require any external sources, which makes the passive approach preferable.

The utilization of inertial forces in curved microchannels has attracted much attention for a wide range of applications [33,34,35,36,37]. Due to the utilization of secondary flow and emerging Dean Drag force, which provide a redistribution of velocity profiles, the lateral positions of particles vary along the cross section of the channel, which can be near the inner wall, the outer wall, or the centerline [16,38]. For example, Ozbey et al. performed a study on symmetrically curved serpentine microchannels in which they utilized Dean flow physics and inertial forces to obtain parallelizable size-based CTC sorting [7]. Hou et al. presented a study related to the separation of CTCs from blood using inertial-based microfluidic separation techniques in a spiral microchannel in which larger cells migrated towards the inner wall [39]. Sun et al. described the size-dependent separation of tumor cells from diluted peripheral whole blood in double-spiral microchannels using secondary flow and Dean Drag force and reported that 92.28% of blood cells were collected at the inner outlet, while 96.77% of tumor cells were collected at the middle outlet [34]. Recently, hybrid microfluidic platforms consisting of both active and passive components have emerged due to their advantages such as multi-target cell processing, wider operational range, high sensitivity, and efficiency [40]. For example, a system with three microfluidic components called CTC-iChip was introduced [41]. The proposed system utilized hydrodynamic cell sorting, inertial focusing, and magnetophoresis in the same platform. RBCs, platelets, CTCs, and WBCs were successfully collected from different outlets. Later, Zhang et al. [42] presented a novel hybrid microfluidic device, in which they obtained particle sorting in a serpentine microchannel by employing inertial and dielectrophoresis forces. The combination of acoustofluidics and inertial forces was performed by Wu et al. in an asymmetric serpentine microchannel for focusing of 10-, 12-, and 15-micron particles and erythrocytes, leukocytes, and cancer cells [43]. They integrated three- dimensional acoustofluidic tweezers (3D-AFT) to a serpentine microchannel. According to their results, they obtained more than 80% purity at the outlet for each size of beads using 3D-AFT-based separation. Similarly, Ren et al. presented a hybrid study in which they utilized standing surface acoustic waves with fluorescence-activated cell sorting in a serpentine microchannel with two outlets [44]. They obtained purities near 90% for HeLa cells without using sheath flow.

The potential of combined hybrid platforms, which utilize surface acoustic waves and inertial forces for cell separation and CTC enrichment, has been studied in several curved microchannels. Yet, the combination of surface acoustic waves with inertial forces, which occurs in a spiral microchannel, has not been adequately revealed. However, implementing surface acoustic waves in passive separation devices with a spiral structure offers a high potential for multi-target cell processing with high-efficiency and high-sensitivity label-free enrichment of circulating tumor cells. By increasing the outlet number of a microchannel and proposing the sorting of cells using SAWs on the generated pressure nodal lines, which occur in the same lateral position of outlets, multi-target processing capabilities can also be accomplished.

Motivated by the abovementioned studies, in this numerical study, we investigated the separation capabilities of hybrid microfluidic devices consisting of active and passive parts. As a passive separation approach, a spiral microchannel structure was utilized to obtain the separation of CTCs based on inertial forces throughout the device. As an active separation approach, a straight microchannel design with a higher rectangular cross-sectional area was added to the end of the spiral microchannel, where interdigital transducers (IDTs) were embedded in the walls of the microchannel reciprocally. In this study, a 3.3 MHz driving frequency was applied to the sidewall of the microchannel from the straight outlet section to represent the transmission of the selected driving frequency into the working fluid in the microchannel. Surface acoustic waves (SAWs) with 454 μm wavelengths produced five nodal lines corresponding to the lateral position of five outlets. According to the physical parameters of the utilized cells, they were trapped and focused on the nodal lines. The separation of CTCs was obtained without using sheath flow. Small footprint of the presented microfluidic platform with two-loop spiral decreased the time needed for the to reach to the outlet, which consequently increased the viability of the cells during the operation. The microchannel, which is presented in this study, with a height of 70 μm and an initial spiral radius of 5 mm, successfully separated three different cell types (CTCs, WBCs, and RBCs) in 0.28 s and 0.187 s at Reynolds numbers of 40 and 65. Cells that exited the passive separation part (spiral section) were sorted according to their diameter using inertial forces. The additional active separation provided enhanced separation of large cells from small ones in the microfluidic platform. Producing five different nodal lines corresponding to the five different outlets allowed trapping the cells, which increased the separation capability. In the presented microfluidic platform, up to five different cell types with various cell diameters can be separated. By implementing the SAWs and nodal lines corresponding to the five outlets, clogging of the cells at the outlet section, which is an important factor in cell separation studies, can also be prevented. Thus, we propose high-throughput, multi-staged, and hybrid microfluidic devices which allow cell separation and the isolation of different cell types according to their sizes. In addition, we present the effect of the microchannel dimensions on the trajectories of the cells for both passive and active separation.

## 2. Theory

Considering the fluid domain in a laminar flow regime in the proposed study, the Reynolds number (Re) is represented as:

Re = (*ρ*UD_h_)/(µ) (1)

where *ρ*, U, D_h_, and µ represent the fluid density (kg/m^3^), the average flow velocity (m/s), the microchannel hydraulic diameter (m), and the fluid dynamic viscosity (kg/ms), respectively.

In microchannels having a rectangular cross section, the hydraulic diameter is represented as:

D_h_ = 2ab/((a + b)) (2)

The magnitude of the formation of Dean vortices and secondary flow in curved microchannels is represented by the dimensionless Dean number [45]:

De = Re√(*δ*) = Re√(D_h_/2R) (3)

where R and *δ* represent the radius of curvature of the microchannel and the ratio of the channel hydraulic diameter to the microchannel radius of curvature, respectively.

A particle or cell in a curved microchannel having a Poiseuille flow experiences inertial Lift force, wall-induced Lift force, Dean Drag force, gravitational force, and buoyancy force. The gravitational force and buoyancy force are assumed to be in equilibrium due to the small dimension of the microchannels. Accordingly, the Dean Drag force (F_De_) [45] and the net inertial force (F_L_) [46] are defined as:

F_De_ = 3πµU_De_ a_p_ (4)

where a_p_ is the particle diameter, and U_De_ is the Dean velocity represented as [45]:

U_De_ = 1.8 × 10^−4^ De^1.63^ (5)

F_L_ = C_L_((ρ(U_max_)^2^(a_p_)^4^)/(D_h_)^2^) (6)

where U_max_ is the maximum fluid velocity, and C_L_ is the Lift coefficient.

The minimum threshold (T), which represents the limitation of successful inertial focusing of the particles, is stated as [43]:

(7) $$T=a_{p}/D_{h}\;\geq \;0.07$$

Accordingly, the ratio of the particle diameter to the channel hydraulic diameter needs to be greater than or equal to 0.07.

In this study, surface acoustic waves (SAWs) were introduced to the microchannel in the straight section before the outlets. Particles experienced an acoustic radiation force (F_r_) and a viscous Drag force (F_d_) (Stokes law) due to the generated pressure distribution in the straight section, which is expressed as [47]:

F_r_ = −(π(*p*_0_)^2^*V_p_β_w_*)/2*λ*) ϕ (*β*, *ρ*) sin(2ky) (8)

ϕ (β, ρ) = ((5ρ_p_ − 2ρ_m_)/(2ρ_p_ + ρ_m_)) − (β_p_/β_m_) (9)

F_d_ = −6πμ*R_pur_* (10)

where *p*_0_, *λ*, *V_p_ ρ_m_*, *ρ_p_*, *β_m_*, *β_p_*, *R_p_*, and *u_r_* represent the acoustic pressure, the acoustic wavelength, the volume of the particle, the density of the medium, the density of the particle, the compressibility of the medium, the compressibility of the particle, the radius of the particle, and the relative velocity of the particle, respectively. ϕ is the acoustic contrast factor, which is used to determine whether the particles will move to a pressure nodal line or to an antinodal line according to its physical properties (volume, compressibility, density). By introducing SAWs, five different pressure nodal lines corresponding to the five outlets were produced, which promoted the movement of the cells towards to the nodal lines. Even though three cell types with different diameters were utilized in this study, the proposed microchannel platform can be implemented for various applications that require a separation of up to five different particles or cell types.

## 3. Material and Methods

### 3.1. Microchannel Design

The microchannel configurations simulated in this study consisted of two-loop spirals with two inlets and five equally distanced 120 μm-wide outlets. The width of each microchannel was 500 μm along the spiral part. The distance between each spiral was fixed at 500 μm. Overall, an aim of the design was to ensure a small footprint.

**Figure 1:**
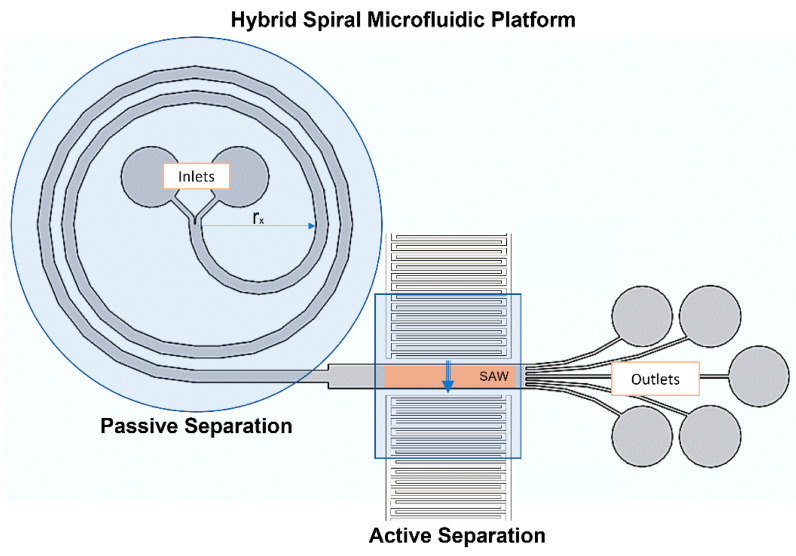
Schematic representation of the hybrid spiral microfluidic platform consisting of passive (spiral) and active (IDTs) components for focusing and separating cells.

Active separation methods require low flow rates, while passive separation occurs at high flow rates [48]. To resolve this issue, the channel geometry was modified according to the requirements of the flow rates of the application. In this regard, an expansion was introduced to the microchannel at the end of the last spiral. A short straight rectangular part (8 mm) decreased the fluid velocity at the active separation component (Figure 1). The width of the microchannel sharply increased to 1 mm at the beginning of this rectangular part.

The radii of the first spiral and the height of the microchannels differed for each microchannel, as presented in Table 1. The S1 and S2 microchannels had a height of 100 μm, while the S3 and S4 microchannels had a height of 70 μm. The radii of the first spiral (r_x_) were set to 10 mm for the S1 and S3 microchannels and to 5 mm for the S2 and S4 microchannels.

Figure 1 shows the schematic representation of the hybrid spiral microfluidic platform, which consisted of passive and active components. In this study, the spiral part served as a passive component and allowed focusing and separating the cells by utilizing the Dean and Lift forces. The straight rectangular section constituted the active component, which enhanced the separation of the cells using surface acoustic waves (SAWs). In Figure 1, input and output IDTs represent the locations of the applied surface acoustic waves schematically.

### 3.2. COMSOL Multiphysics Modeling

Passive separation, which utilized Lift and Drag forces in the spiral microchannel, was performed using the Laminar Flow and Particle Tracing Module for Fluid Flow Physics of the Software COMSOL Multiphysics 5.5 [49]. In the simulations, white blood cells (WBCs), red blood cells (RBCs), and circulating tumor cells (CTCs) were defined by considering them as particles with different diameters and a density (*ρ_p_*) of 1050 kg/m^3^. WBCs, RBCs, and CTCs were represented as particles with a diameter of 9 μm, 6 μm, and 20 μm, respectively.

The inlet of the microchannel was arranged in such a way that the particles were released from one common inlet. The physical model for the Laminar Flow Module was chosen as incompressible flow.

To include the effect of the actual shear gradient force which acted on the particles, the discretization was set as P2 + P2. By defining different inlet velocities, the Reynolds number at which efficient separation was obtained was found. With a stationary study, the Laminar Flow Physics was explored for each microchannel presented in Table 1 at Reynolds numbers of 40 and 65. The governing Navier–Stokes equations for an incompressible and steady laminar flow are as follows:

(11) $$\rho \left(u\cdot \nabla \right)u=\nabla \cdot \left[-pI+\mu (\nabla u+{\left(\nabla u\right)}^{T}\right]$$

(12) ρ∇·u=0

where u and p represent the velocity vector and the pressure, respectively. The dynamic viscosity and density were implemented from COMSOL Multiphysics 5.5 as a selection of material, i.e., water, liquid.

After finding the velocity field from the stationary study, the Particle Tracing Module for Fluid Flow Physics was utilized to introduce different cell types. The Newtonian formulation for particle release and propagation and bounce wall condition was chosen. Due to low Reynolds numbers, the Drag Law was considered as the Stokes Law, while the Lift Force was set to the wall-induced Lift Law. For both Laminar Flow and Particle Tracing Module for Fluid Flow Physics, a physics-controlled mesh with normal element size was used for the microchannels. With a time-dependent study, particle trajectories were obtained so that the cells were separated according to their diameter by the effects of the Drag and wall-induced Lift forces in the spiral microchannels. The governing equations for particle tracing for fluid flow are as follows:

(13) $$F_{t}=\frac{d\left(m_{p}v\right)}{\mathrm{dt}}$$

where $F_{t}$, $m_{p}$, and v are the total force, the particle mass, and the particle velocity, respectively.

**Figure 2:**
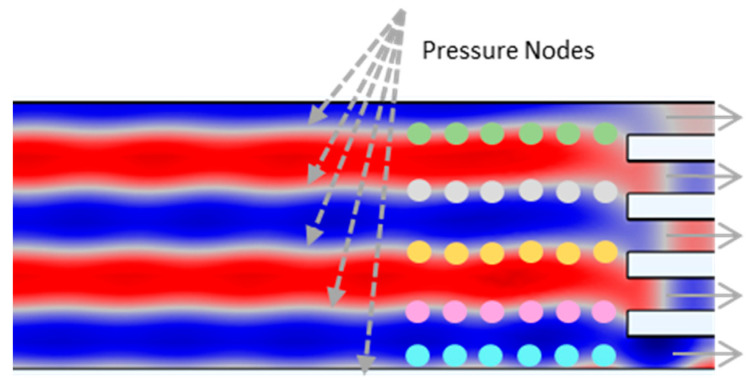
Acoustic pressure distribution in the straight outlet section. Due to the applied SAWs, five nodal pressure lines corresponding to the lateral position of the outlets were generated.

Active separation, which was induced by pressure distribution due to the applied SAWs, was also simulated using the Pressure Acoustics, Frequency Domain, and Particle Tracing Module for Fluid Flow Physics of the Software COMSOL Multiphysics 5.5. Since SAWs were applied in the straight rectangular section of the spiral microchannels before the particles were split into five outlets (Figure 2), the straight rectangular part was isolated and studied separately. In the Pressure Acoustics Module, the fluid model was set to linear elastic. The Drag Law was considered as the Stokes Law. The particle type was chosen as solid and implemented for the radiation force model. The length of the transducer (*l*) was defined as 5 mm. The driving frequency (f) was set to 3.3 MHz. Thus, assuming the speed of sound in water, c, as 1500 m/s, the wavelength of the SAWs (λ) was 454 μm, which produced five nodal lines in the microchannel and corresponded to the lateral position of the five outlets (Figure 2). After obtaining the acoustic pressure distribution and sound pressure level in the microchannel by using a frequency-domain study, three different particles with different diameters representing RBCs, WBCs, and CTCs were defined. We chose a density of 1050 kg/m^3^, based on the density of the cells, and the particles were released from the inlet. The location of the initial position of the particles was mesh-based with zero initial velocity before applying the SAWs. As a result of the generated pressure distribution, the particles experienced the acoustic radiation forces and viscous Drag forces. The particle trajectories were determined using a time-dependent study. The governing equations for Frequency-Domain Pressure Acoustics are as follows:

(14) $$Q_{m}=\nabla =\left(-\frac{1}{\rho_{c}}\left(\nabla p_{t}-q_{d}\right)\right)-\frac{k_{eq}^{2}p_{t}}{\rho_{c}}$$

(15) $$p_{t}=p+p_{b}$$

(16) $$k_{eq}^{2}={\left(\frac{\omega }{c_{c}}\right)}^{2}-k_{z}^{2}$$

where $Q_{m}$, $p_{c}$, $p_{t}$, $q_{d}$, $p_{b}$, ω, $c_{c}$, and *k* represent the monopole domain source, the complex density, the total acoustic pressure, the dipole domain source, the background pressure, the angular velocity, the speed of sound, and the wave number, respectively.

For the Laminar Flow Physics, a no-slip boundary condition was applied on the channel walls. For the inlet boundary condition, normal inflow velocity was applied at the studied Reynolds numbers of 40 and 65 for each microchannel, whereas a zero-pressure boundary condition was imposed for the outlets.

In the Particle Tracing Module for Fluid Flow Physics, a bounce boundary condition was applied on the channel walls. In the passive separation study, a freeze boundary condition was implemented on the outlets of the channel walls to enable the detection of the state of the cells at the outlet.

It should be noted that for the active separation analysis, cells with different diameters and zero initial velocities were simulated in the applied acoustic field to demonstrate the effect of the 3.3 MHz driving frequency on a large number of particles. The active separation analysis was conducted independently of the passive separation analysis.

## 4. Results and Discussion

### 4.1. Passive Separation

**Figure 3:**
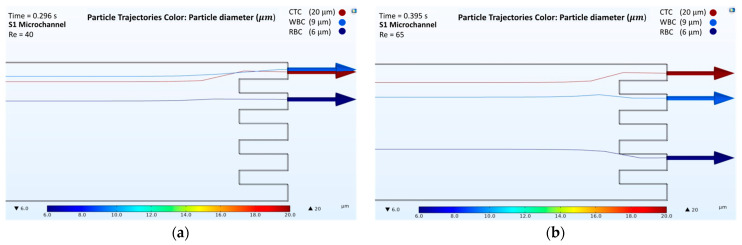
Numerical results of the cell separation study in the S1 microchannel. CTC, WBC, and RBC cells are represented by dark red, light blue, and dark blue colors, respectively: (**a**) Cell distribution at the outlet of the S1 microchannel at a Reynolds number of 40; (**b**) Cell distribution at the outlet of the S1 microchannel at a Reynolds number of 65.

**Figure 4:**
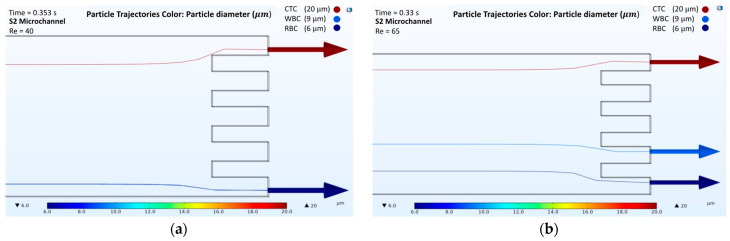
Numerical results of the cell separation study in the S2 microchannel. CTC, WBC, and RBC cells are represented by dark red, light blue, and dark blue colors, respectively: (**a**) Cell distribution at the outlet of the S2 microchannel at a Reynolds number of 40; (**b**) Cell distribution at the outlet of the S2 microchannel at a Reynolds number of 65.

**Figure 5:**
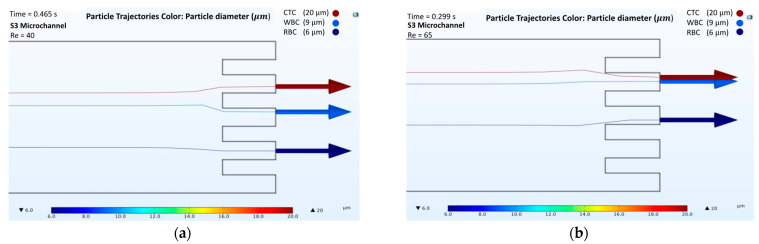
Numerical results of the cell separation study in the S3 microchannel. CTC, WBC, and RBC cells are represented by dark red, light blue, and dark blue colors, respectively: (**a**) Cell distribution at the outlet of the S3 microchannel at a Reynolds number of 40; (**b**) Cell distribution at the outlet of the S3 microchannel at a Reynolds number of 65.

**Figure 6:**
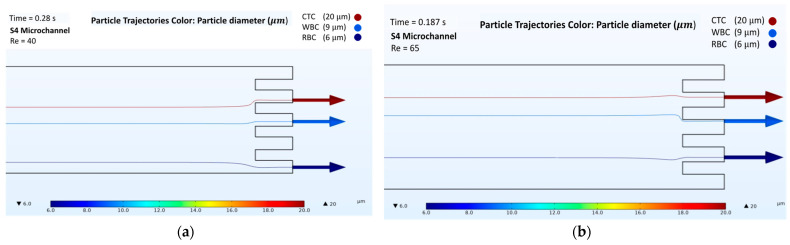
Numerical results of the cell separation study in the S4 microchannel. CTC, WBC, and RBC cells are represented by dark red, light blue, and dark blue colors, respectively: (**a**) Cell distribution at the outlet of the S4 microchannel at a Reynolds number of 40; (**b**) Cell distribution at the outlet of the S4 microchannel at a Reynolds number of 65.

The results obtained from the numerical simulations of passive separation in the spiral microchannel illustrated that the separation of cells could be obtained from different outlets at different Reynolds numbers. For the analysis, four microchannels with a two-loop spiral structure with a straight rectangular section were used. The presented microchannels had different channel heights and radii of the first spirals, as shown in Table 1. All microchannels were considered at Reynolds numbers of both 40 and 65. According to the behavior of the cells in different velocity fields, the trajectories of RBCs, WBCs, and CTCs were obtained (Figures S1–S8 for velocity field distributions, in Supplementary Materials). Figure 3, Figure 4, Figure 5 and Figure 6 show the passive cell separation results of the S1, S2, S3, and S4 microchannels, respectively.

Due to the a/D_h_ ratio represented in Equation (7), cells with a diameter greater than or equal to 11.7 μm were affected by inertial forces in the S1 and S2 microchannels. For the S3 and S4 microchannels, this diameter was 8.6 μm. Particles with larger diameters were exposed to greater Dean Drag forces. CTCs with a diameter of 20 μm were more affected by the Dean Drag force due to their larger diameter compared to the other particles, which resulted in focusing the CTC cell line near the inner wall of each presented microchannel.

Figure 3a and Figure 4a show the trajectories of the cells at a Reynolds number of 40 in the S1 and S2 microchannels, respectively. At a Reynolds number of 40, the S1 and S2 microchannels were not capable of providing sufficient separation. However, an increase in the inlet velocity caused the dominance of the Dean Drag and net inertial forces along the microchannels, which consequently affected the trajectories of the cells. Figure 3b and Figure 4b show that CTC, WBC, and RBC cells were collected from the different outlets at a Reynolds number of 65 for the S1 and S2 microchannels, respectively. However, due to the shorter length of the S2 microchannel, the time needed for the cells to be collected from the outlets, 0.33 s, was shorter than the time needed for cell collection from the S1 microchannel, that was 0.395 s.

Figure 5 presents the results of the cell trajectories in the S3 microchannel. At Re = 40, CTC, WBC, and RBC were collected in 0.465 s from the second, third, and fourth outlets, respectively (Figure 5a). However, an increase in the inlet velocity caused a larger Dean Drag force on WBC, which forced them to move towards the second outlet at Re = 65 (Figure 5b). Thus, an efficient separation of the cells was not achieved with the S3 microchannel at a Reynolds number of 65.

In the S4 microchannel, at both Reynolds numbers (Re = 40 and Re = 65), the three kinds of cells (CTC, WBC, and RBC) exited the microchannel from different outlets (Figure 6a,b). CTCs, which had a diameter of 20 μm, were affected more by the Dean Drag force due to their larger diameter compared to those of the other cells (WBCs and RBCs), which resulted in focusing the CTCs near the inner wall of the microchannel. Thus, CTCs were collected from the second outlet at Reynolds numbers of 40 and 65. WBCs, which are smaller than CTCs and larger than RBCs, were less affected by the Dean Drag force and exited the microchannel from the third outlet. RBCs exited the microchannel from the fifth and fourth outlets at Re = 40 and Re = 65, respectively. The duration of the cell collecting times at Reynolds numbers of 40 and 65 were 0.28 and 0.187 s, respectively, for the S4 microchannel. Overall, the S4 microchannel was superior to the other microchannels as it provided a faster separation of the cells in 0.187 s at Re = 65.

### 4.2. Active Separation

In the hybrid microfluidic platform, we proposed focusing of the cells prior to their exposure to SAWs in acoustofluidic separation (active separation), which was obtained by using the spiral microchannel structure (passive separation). However, to increase the sensitivity and separation efficiency of the device, an active separation technique was realized by introducing SAWs. The combination of inertial focusing with SAWs facilitated the trapping of the particles along the nodal lines leading to the five outlets. The proposed two-step approach is promising as a means of increasing the sensitivity of separation, as indicated by the excellent localization of the 20 μm particles along the nodal lines, whereas the 6 μm and 9 μm particles experienced clear dispersion and mixing.

SAWs with 454 μm wavelengths were introduced from the sidewall of the microchannels. The simulation parameters related to SAW study are represented in Table 2. Five different pressure nodal lines were produced in the same lateral position as the outlets. It has been reported in the literature that these multiple pressure nodal line formations along microchannels have advantages for separation applications since they provide significantly improved efficiency and sensitivity compared to SAW-based separation devices with single pressure nodal or antinodal lines [43].

**Figure 7:**
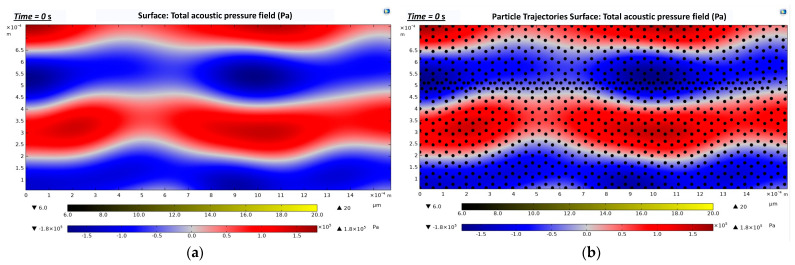
Total acoustic pressure field (zoom-in images that show three pressure nodal lines occurring in the straight rectangular section): (**a**) Formation of an acoustic pressure field along the straight rectangular section of the microchannel. The blue and red areas represent the pressure antinodal lines. The white areas represent the pressure nodal lines; (**b**) Cells trajectories at t = 0 s. The cells were set to zero initial velocity.

Figure 7a presents the acoustic pressure field (Pa) distribution along the straight rectangular section of the microchannel. A maximum acoustic pressure of 1.8 × 10^5^ Pa (dark red) and a minimum acoustic pressure of −1.8 × 10^5^ Pa (dark blue) were generated. The areas in the Figure 7 indicated in blue and red represent the pressure antinodal lines, whereas the white areas represent the pressure nodal lines. Three kinds of cells with different diameters (CTCs, WBCs, and RBCs) were located with zero initial velocity at t = 0 s (Figure 7b).

**Figure 8:**
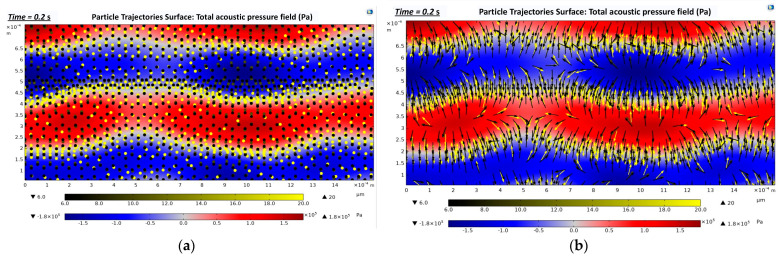
Trajectories of the cells during 0.2 s of exposure to a 3.3 MHz driving frequency (zoom-in images that show three pressure nodal lines occurring in the straight rectangular section): (**a**) Location of the cells in t = 0.2 s along the straight rectangular section of the microchannel; (**b**) Motions of the cells while they move towards the pressure nodal lines in t = 0.2 s.

Figure 8a represents the location of the cells when they were exposed to a 3.3 MHz driving frequency for 0.2 s. As SAWs were applied from the sidewall of the microchannel, the cells were exposed to the acoustic radiation force. They lined up on the pressure nodal lines, as described in Figure 7a (white areas). In Figure 8b, the arrows represent the motions of the cells while they moved towards the pressure nodal lines.

**Figure 9:**
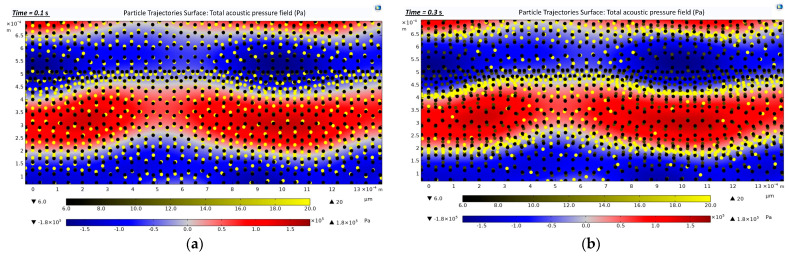

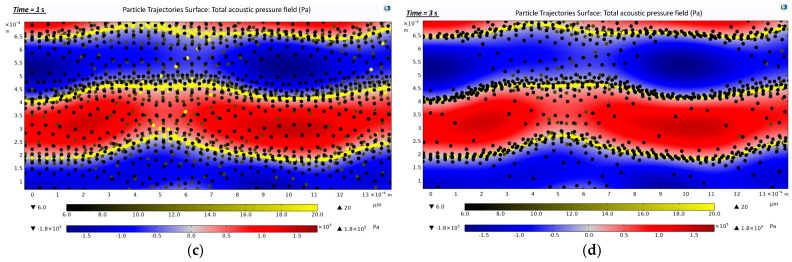
Distribution of the cells subjected to the acoustic pressure field along the straight rectangular section of the microchannel serving as an active separation in the hybrid device (zoom-in images that show three pressure nodal lines occurring in the straight rectangular section): (**a**) Cell trajectories at t = 0.1 s; (**b**) Cell trajectories at t = 0.3 s; (**c**) Cell trajectories at t = 1 s; (**d**) Cell trajectories at t = 3 s.

Particles with larger diameters experienced larger acoustic radiation forces and Drag forces. This led to a clear positioning of larger cells (CTCs) along the nodal lines, while smaller cells (WBCs, RBCs) displayed a less accurate distribution (Figure 9). In Figure 9, CTCs with larger diameters (20 μm) are in light yellow and were more affected by SAWs, sorting on the pressure nodal lines. The CTCs moved towards the pressure nodal lines in 0.1 s upon exposure to 3.3 MHz driving frequency (Figure 9a). At t = 0.3 s, the CTCs were trapped on the pressure nodal lines (Figure 9b). However, smaller particles (WBCs and RBCs) required more time to be sorted in the pressure nodal lines (Figure 9c,d).

## 5. Conclusions

This study presents a multi-stage hybrid microfluidics platform that utilizes inertial and acoustic radiation forces for particle/CTC separation studies. The spiral microchannel structure allowed focusing different kinds of cells using the Dean Drag force and Lift forces. These cells later experienced acoustic radiation forces that were applied along the straight rectangular section of the microchannel. The SAWs wavelength was set to 454 μm. Five pressure nodal lines, which were in the same lateral position of the outlets, were produced. Thus, the cells, which were not lined up after exiting the spiral microchannel, were able to be captured and trapped on the pressure nodal lines depending on their diameters.

A microchannel, with a height of 70 μm and an initial spiral radius of 5 mm, successfully separated three different cell types (CTCs, WBCs, and RBCs) in 0.28 s and 0.187 s at Reynolds numbers of 40 and 65, respectively.

In the light of the findings of this study, we propose that microchannels with these features, which are capable of separating cells at both studied Reynolds numbers and provided the fastest cell separation in this study, can be fabricated as polydimethylsiloxane (PDMS) microchannels, and IDT structures (length, finger distance, thickness) can be designed and coupled with such PDMS microchannels.

## Figures and Tables

**Table 1:** Common geometrical parameters and dimensions of the microchannels which are named as S1, S2, S3, and S4.

Channel Name	Channel Width at the Spiral Part (μm)	Channel Width at the Straight Part (mm)	Channel Height (μm)	Radius of the First Spiral (r_x_) (mm)
S1	500	1	100	10
S2			100	5
S3			70	10
S4			70	5

**Table 2:** Simulation parameters related to the SAW study.

Description	Expression	Value
Driving frequency	f	3.3 [MHz]
Speed of sound	c	1500 [m/s]
Wavelength	λ	454 [μm]
Transducer length	l	5 [mm]
Particle density	*ρ_p_*	1050 [kg/m^3^]

## Data Availability

Not applicable.

## Supplementary Materials

The following supporting information can be downloaded at: https://www.mdpi.com/article/10.3390/bios12030171/s1, Figure S1: The results of the cell trajectories and velocity field in the S1 microchannel at Reynolds number of 40. CTC, WBC, and RBC were represented by dark red, light blue, and dark blue colors, respectively; Figure S2: The results of the cell trajectories and velocity field in the S1 microchannel at Reynolds number of 65. CTC, WBC, and RBC were represented by dark red, light blue, and dark blue colors, respectively; Figure S3: The results of the cell trajectories and velocity field in the S2 microchannel at Reynolds number of 40. CTC, WBC, and RBC were represented by dark red, light blue, and dark blue colors, respectively; Figure S4: The results of the cell trajectories and velocity field in the S2 microchannel at Reynolds number of 65. CTC, WBC, and RBC were represented by dark red, light blue, and dark blue colors, respectively; Figure S5: The results of the cell trajectories and velocity field in the S3 microchannel at Reynolds number of 40. CTC, WBC, and RBC were represented by dark red, light blue, and dark blue colors, respectively; Figure S6: The results of the cell trajectories and velocity field in the S3 microchannel at Reynolds number of 65. CTC, WBC, and RBC were represented by dark red, light blue, and dark blue colors, respectively; Figure S7: The results of the cell trajectories and velocity field in the S4 microchannel at Reynolds number of 40. CTC, WBC, and RBC were represented by dark red, light blue, and dark blue colors, respectively; Figure S8: The results of the cell trajectories and velocity field in the S4 microchannel at Reynolds number of 65. CTC, WBC, and RBC were represented by dark red, light blue, and dark blue colors, respectively; Video S1: The video shows the particle trajectories in the velocity field in the S4 microchannel at a Reynolds number of 65. CTC, WBC, and RBC cells are identified by color. Accordingly, CTC, WBC, and RBC cells are shown in dark red, light blue, and dark blue color, respectively. Video S2: The video shows the particle trajectories in the applied acoustic pressure fields for 3 s. The maximum and minimum acoustic pressures that were formed in the microchannel are shown in the legend. The maximum and minimum of approximately 1.8 × 10^5^ Pa and −1.8 × 10^5^ Pa acoustic pressure fields are shown in dark red and dark blue colors, respectively. CTC, WBC, and RBC cells are shown in yellow shades. Accordingly, CTC, WBC, and RBC cells are ranged from light yellow to dark yellow, respectively.
