# Supplementary material for: A Hybrid Spiral Microfluidic Platform Coupled with Surface Acoustic Waves for Circulating Tumor Cell Sorting and Separation: A Numerical Study
Source: Biosensors (Basel). 2022 Mar 11;12(3):171. doi: 10.3390/bios12030171 (PMC8946654; doi:10.3390/bios12030171)
Supplement: Supplementary file 1 [file biosensors-12-00171-s001.zip › biosensors-1600585-supplementary.pdf]

biosensors

MDPI

Supplementary Information

A Hybrid Spiral Microfluidic Platform Coupled with Surface Acoustic Waves for Circulating Tumor Cell Sorting and Separation: A Numerical Study

Rana Altay [1], Murat Kaya Yapici [1,2] and Ali Koşar [1,2,*]

[1] Faculty of Engineering and Natural Sciences, Sabanci University, Istanbul 34956, Turkey; raltay@sabanciuniv.edu (R.A.); mkyapici@sabanciuniv.edu (M.K.Y.)
[2] Center of Excellence for Functional Surfaces and Interfaces for Nano-Diagnostics, Faculty of Engineering and Natural Sciences, Sabanci University, Istanbul 34956, Turkey
* Correspondence: kosara@sabanciuniv.edu

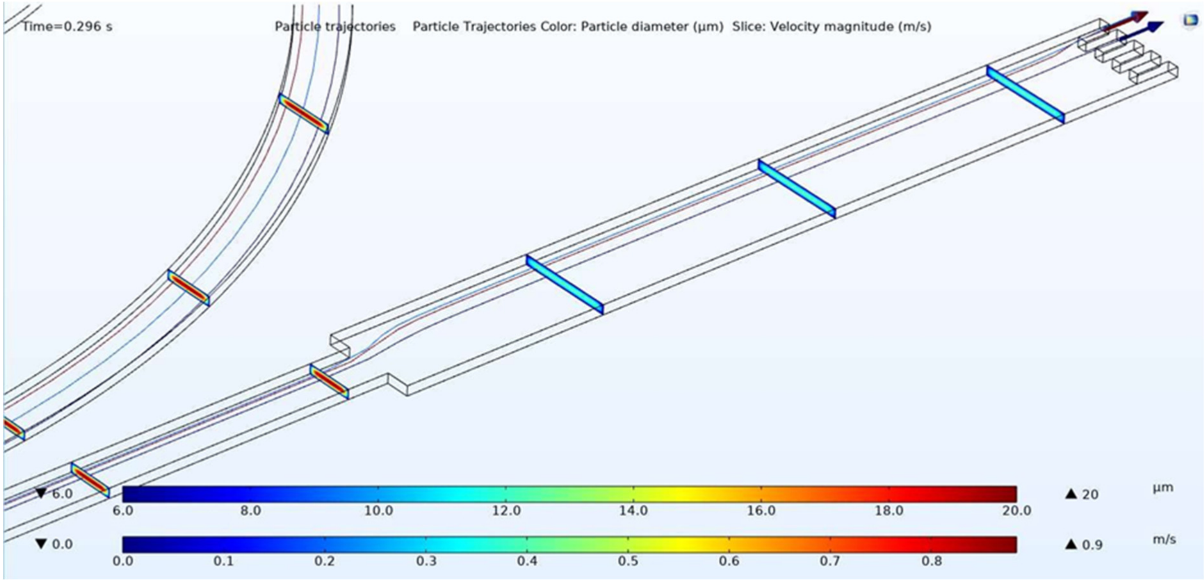

Figure S1. The results of the cell trajectories and velocity field in the S1 microchannel at Reynolds number of 40. CTC, WBC, and RBC were represented by dark red, light blue, and dark blue colors, respectively.

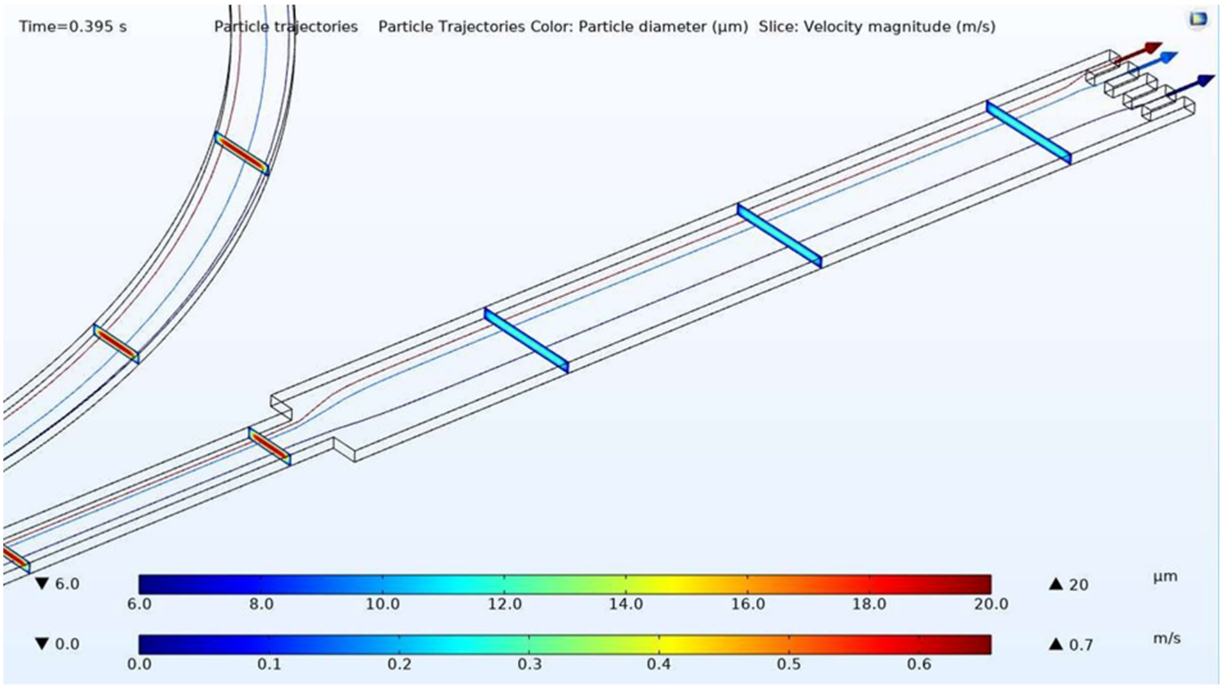

Figure S2. The results of the cell trajectories and velocity field in the S1 microchannel at Reynolds number of 65. CTC, WBC, and RBC were represented by dark red, light blue, and dark blue colors, respectively.

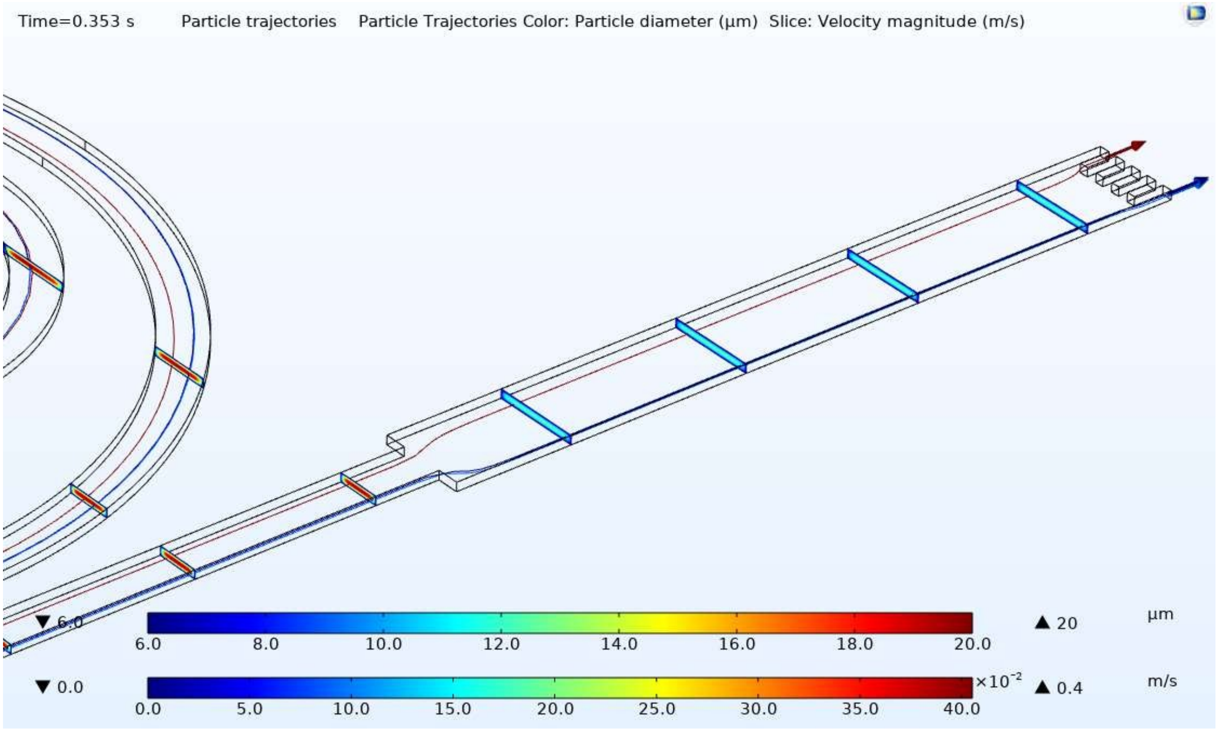

Figure S3. The results of the cell trajectories and velocity field in the S2 microchannel at Reynolds number of 40. CTC, WBC, and RBC were represented by dark red, light blue, and dark blue colors, respectively.

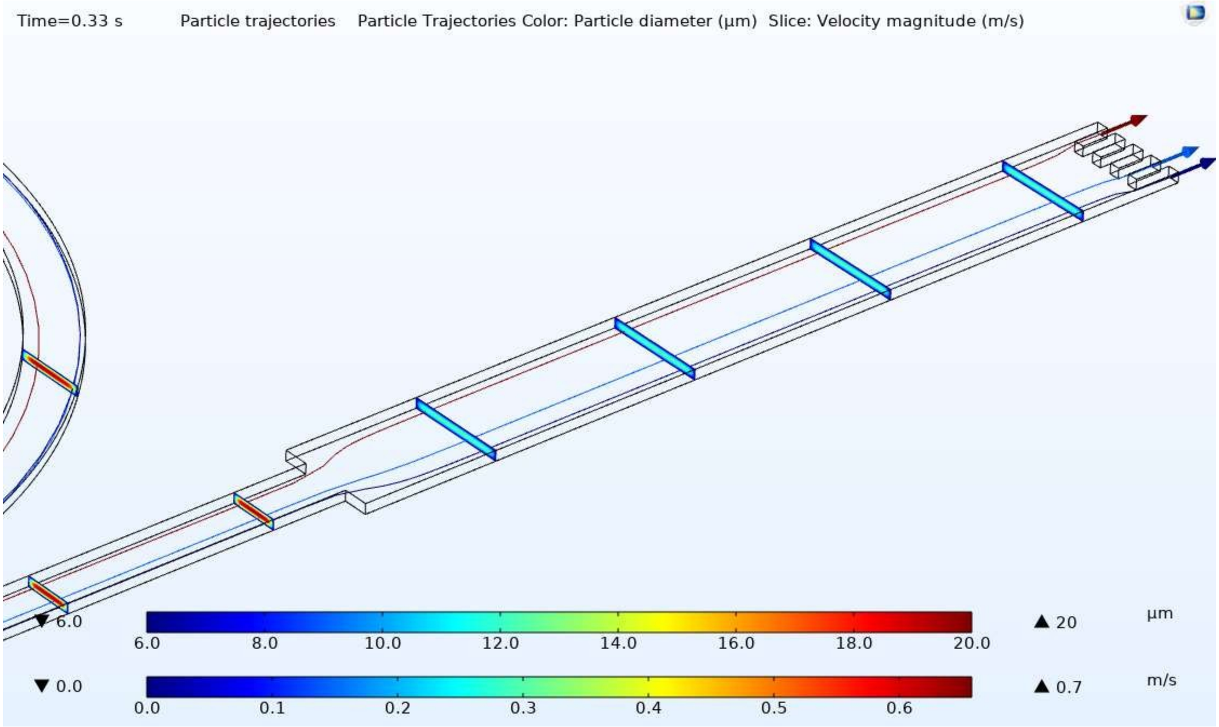

Figure S4. The results of the cell trajectories and velocity field in the S2 microchannel at Reynolds number of 65. CTC, WBC, and RBC were represented by dark red, light blue, and dark blue colors, respectively.

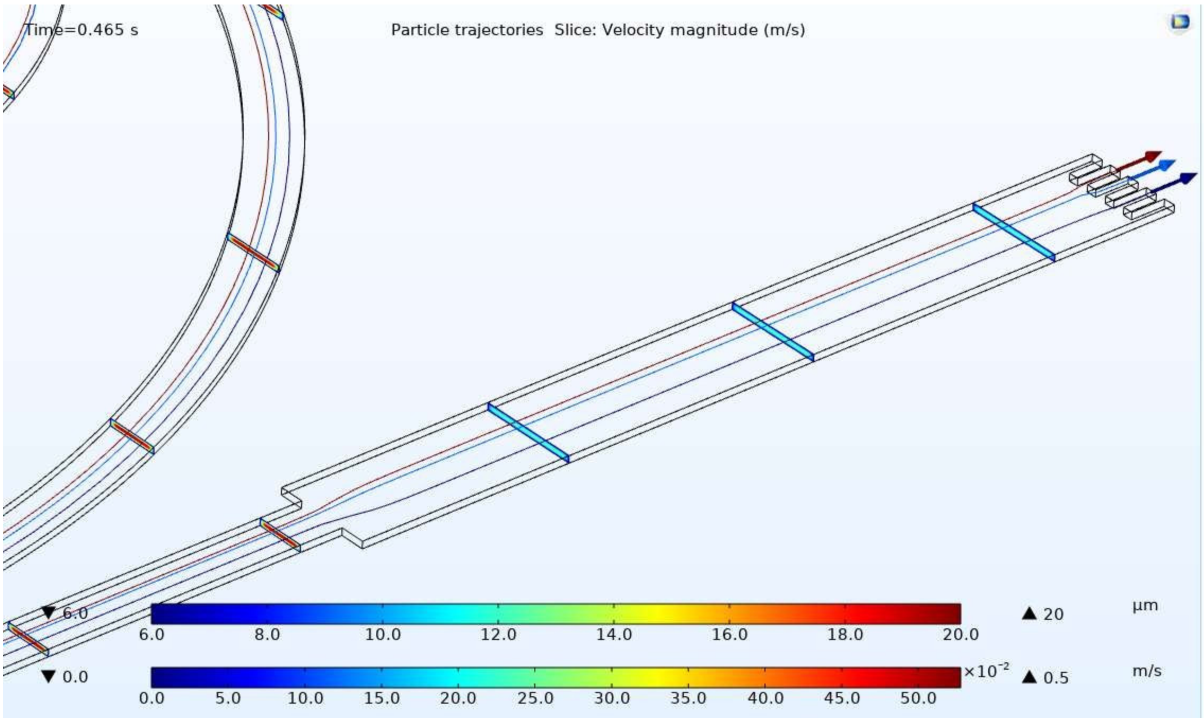

Figure S5. The results of the cell trajectories and velocity field in the S3 microchannel at Reynolds number of 40. CTC, WBC, and RBC were represented by dark red, light blue, and dark blue colors, respectively.

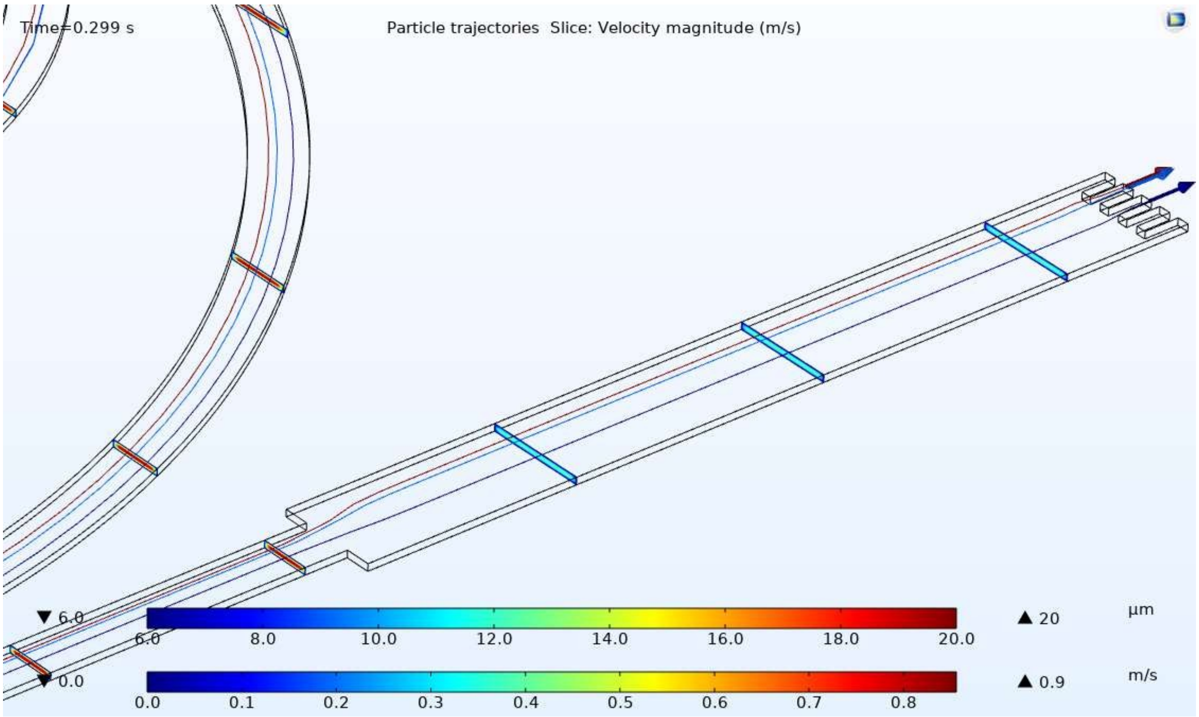

Figure S6. The results of the cell trajectories and velocity field in the S3 microchannel at Reynolds number of 65. CTC, WBC, and RBC were represented by dark red, light blue, and dark blue colors, respectively.

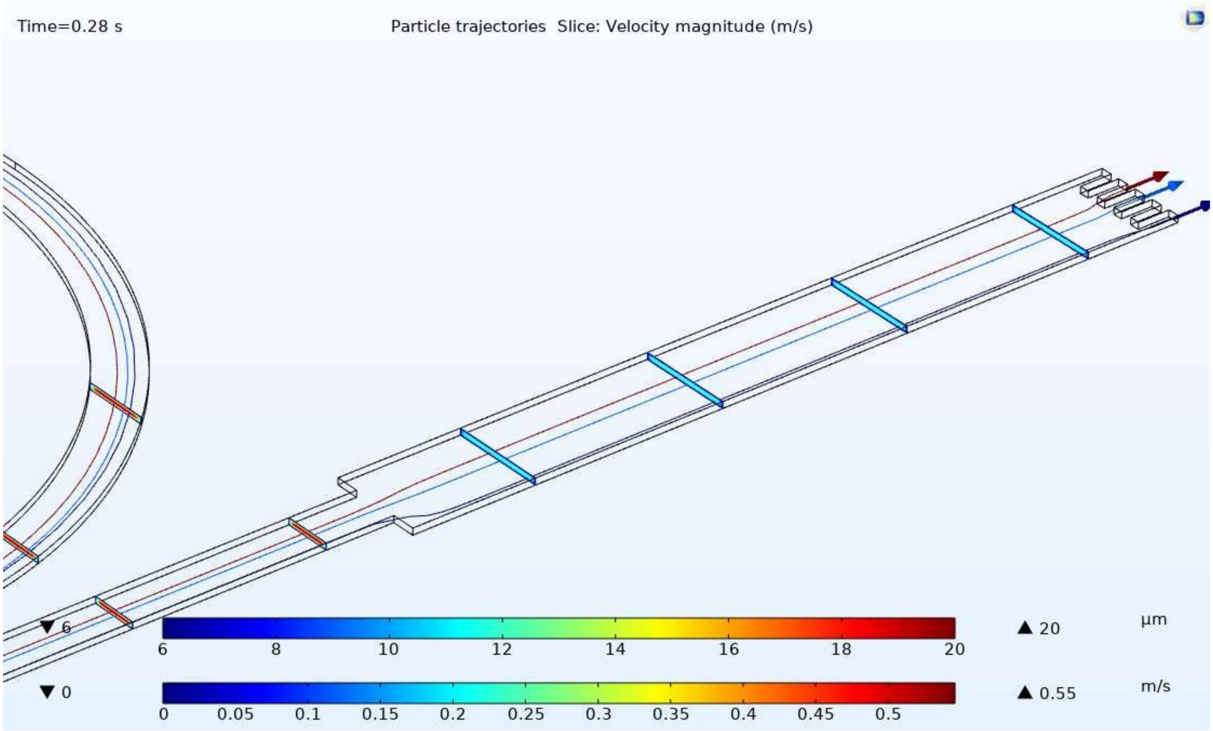

Figure S7. The results of the cell trajectories and velocity field in the S4 microchannel at Reynolds number of 40. CTC, WBC, and RBC were represented by dark red, light blue, and dark blue colors, respectively.

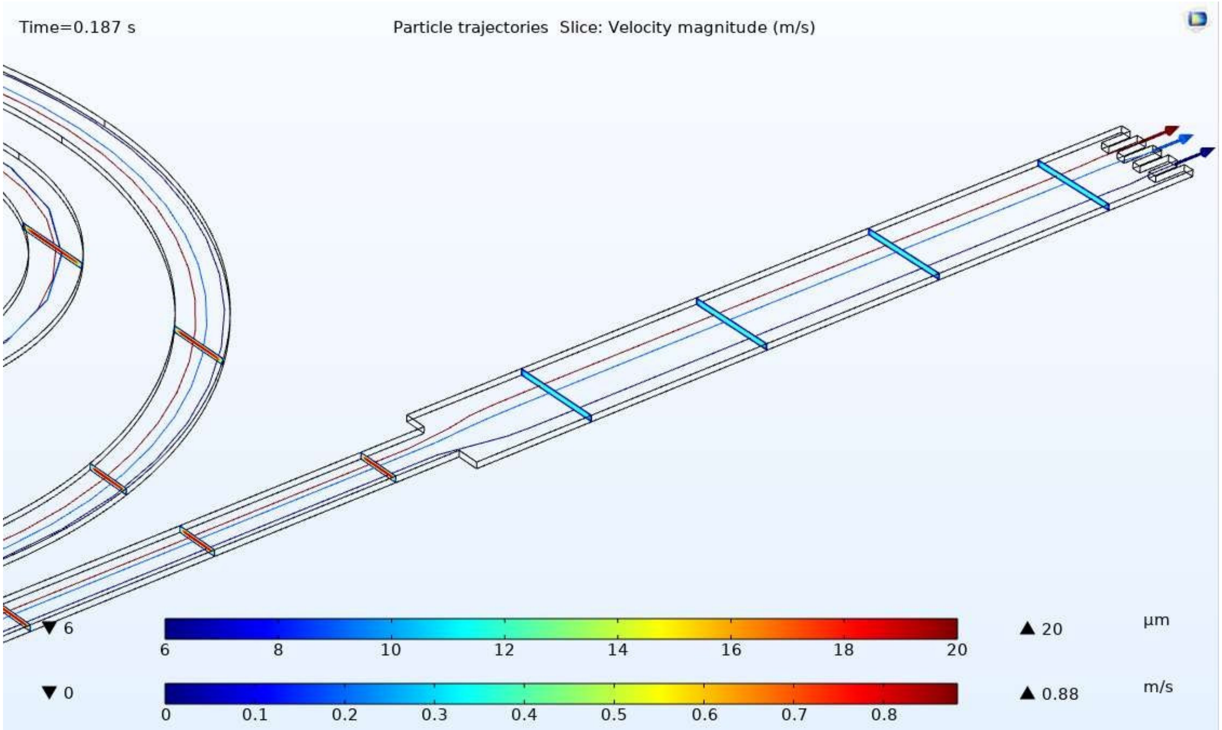

Figure S8. The results of the cell trajectories and velocity field in the S4 microchannel at Reynolds number of 65. CTC, WBC, and RBC were represented by dark red, light blue, and dark blue colors, respectively.

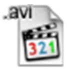

Video S1.avi.

Video S1. The video shows the particle trajectories in the velocity field in the S4 microchannel at Reynolds number of 65. The CTC, WBC, and RBC are represented by the color legend. CTC, WBC, and RBC are shown by dark red, light blue, and dark blue colors, respectively.

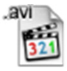

VideoS2.avi.

Video S2. The video shows the particle trajectories in the applied acoustic pressure fields for 3 seconds. The maximum and minimum acoustic pressures, which were formed in the microchannel, are shown in the legend. Themaximum and minimum of approximately 1.8 x 10^5 Pa and - 1.8 x 10^5 Pa acoustic pressure fields are shown with the dark red and dark blue colors, respectively. CTC, WBC, and RBC are represented by the yellow color legend. CTC, WBC, and RBC are ranged from light yellow to dark yellow colors.
